# Comparative Effects of Hydropriming and NaHS-Priming on Salinity Tolerance in *Brassica napus* L. Seedlings

**DOI:** 10.3390/plants15040551

**Published:** 2026-02-10

**Authors:** Faezeh Bazvand, Łukasz Wojtyla, Małgorzata Adamiec, Małgorzata Garnczarska

**Affiliations:** 1 Department of Plant Production Engineering and Genetics, Faculty of Agriculture, Lorestan University, Khorramabad 68151-44316, Iran; 2 Department of Plant Physiology, Institute of Experimental Biology, Faculty of Biology, Adam Mickiewicz University, Poznan, ul. Uniwersytetu Poznanskiego 6, 61-614 Poznan, Polandmsolin@amu.edu.pl (M.A.)

**Keywords:** salinity stress, hydrogen sulfide, priming

## Abstract

Salinity stress significantly restricts crop productivity by impairing germination and early seedling growth through osmotic, ionic, and oxidative damage. The present study evaluated whether sodium hydrosulfide (NaHS) priming confers greater protection than hydropriming in *B. napus* (*Brassica napus* L). Seeds were primed with water, 0.1 mM NaHS, or 10 mM NaHS and then germinated under saline conditions. Parameters measured included germination rate, root length, endogenous hydrogen sulfide (H_2_S) content, antioxidant capacity, oxidative stress markers, and membrane integrity. Salinity reduced germination to 30% and root length to 1.6 mm in unprimed seeds. Both 0.1 mM and 10 mM NaHS priming produced more substantial improvements in these parameters compared to hydropriming. The most pronounced effect was observed with 10 mM NaHS, which after 48 h increased germination to 100% and root length to 30 mm. Furthermore, 10 mM NaHS priming most effectively elevated endogenous H_2_S levels, enhanced antioxidant capacity (60.43 µM TE/g FW at 24 h), reduced hydrogen peroxide (H_2_O_2_) (2.04 nmol g^−1^ FW at 48 h) and thiobarbituric acid reactive substances (TBARS) (0.008 mmol g^−1^ FW at 48 h), and preserved membrane integrity by limiting Evans staining and electrolyte leakage. In summary, NaHS priming provided substantially greater mitigation of salinity-induced damage than hydropriming in *B. napus*.

## 1. Introduction

In recent years, considerable research has focused on understanding the impact of salinity stress on plant physiological processes. This interest arises from the significant effect of this abiotic stress on plant growth and development. Currently, soil salinization affects over one billion hectares of agricultural land worldwide [1], resulting in annual economic losses of billions of dollars and presents a critical challenge to global food security. Therefore, developing and implementing effective strategies to mitigate salinity stress is essential for ensuring sustainable agricultural productivity. The overaccumulation of ions in soil leads to osmotic stress and reduces a plant’s ability to absorb water and mineral salts. Ion overaccumulation also triggers the formation of significant amounts of reactive oxygen species (ROS)—like superoxide anion (O_2_^−^•), hydroxyl radical (•OH), and hydrogen peroxide (H_2_O_2_). This, in turn, results in oxidative damage to cellular components such as DNA, lipids, and proteins [2], and consequently impairs plant growth and development [3]. To mitigate these effects, plants activate pathways that lead to the upregulation of antioxidant enzymes, such as superoxide dismutase (SOD), catalase (CAT), and ascorbate peroxidase (APX) [3]. Beyond oxidative stress, salinity induces ion toxicity, which reduces the activity of many enzymes and disrupts membrane integrity. It also further interferes with the uptake of essential nutrients, which are already reduced due to osmotic stress. To counteract this, plants activate mechanisms that include osmotic changes and ion compartmentalization, for example, the sequestration of Na^+^ into vacuoles stress [4].

Seed priming is an effective and low-cost technique for mitigating the adverse effects of salinity stress on plants. It involves the pre-sowing treatment of seeds with various physical, chemical, or biological agents. These stimuli—such as water, salts, plant growth regulators, antioxidants, osmoprotectants, and beneficial microbes—initiate metabolic processes that prepare seeds for rapid and uniform germination under stress conditions [5,6]. Priming induces physiological and molecular changes that collectively enhance a plant’s ability to tolerate salinity stress. At the physiological level, priming enhances seed hydration and activates metabolism, leading to faster germination [7]. At the molecular level, it improves energy metabolism, increases antioxidant enzyme activity, and promotes the accumulation of osmoprotectants, such as proline and soluble sugars, which help maintain cellular homeostasis under stress [8]. It also upregulates stress-responsive and germination-related genes while downregulating those involved in dormancy induction, stimulating DNA repair mechanisms and cell cycle progression, further preparing the seed for rapid growth [9]. Additionally, priming alters the internal plant hormone balance, increasing gibberellin levels while decreasing abscisic acid, and activates signaling molecules like reactive oxygen species (ROS) and nitric oxide (NO), which play crucial roles in stress perception and response. These changes create a form of “stress memory”, enabling primed seeds to better withstand adverse saline conditions during germination and early seedling development [10].

The hydrogen sulfide H_2_S has shown promising potential as a seed priming agent in enhancing plant tolerance to abiotic stress conditions [11]. Recognized as the third gasotransmitter after carbon monoxide (CO) and NO, H_2_S plays a crucial role in various biochemical and physiological processes, particularly in plant responses to environmental stressors. H_2_S mitigates the effects of salinity stress by maintaining ionic homeostasis as well as by modulating both H_2_S metabolism and oxidative stress responses [12].

Previous studies have demonstrated the beneficial effects of H_2_S application in several plant species. H_2_S treatment was proven to enhance antioxidant defense mechanisms and reduce lipid peroxidation in *Brassica juncea* [13], *Phaseolus vulgaris* [14], *Lycium barbarum* [15], and *Triticum aestivum* [16]. In *Cucumis sativus*, H_2_S treatment significantly increased endogenous H_2_S levels by modulating both H_2_S-synthetic and degradative enzymes. Under salinity stress, this rise in endogenous H_2_S not only enhanced the plant’s internal H_2_S pool but also contributed to maintaining ion homeostasis through regulation of the salt overly sensitive signaling pathway and to reducing ROS accumulation, thereby alleviating oxidative damage [17]. Additionally, seed priming with sodium hydrogen sulfide (NaHS), a commonly used donor of H_2_S, enhanced germination under stress conditions in *Nicotiana tabacum* [18].

*Brassica napus* L. (rapeseed) is valued for its high oil and protein content, making it one of the most important oilseed crops [19]. Beyond its nutritional value, it is also widely used in the cosmetic and pharmaceutical industries due to its unique biochemical properties [20]. Rapeseed (*Brassica napus* L). exhibits moderate tolerance to salinity, and high ion concentrations in the soil can significantly impair its growth and yield [21]. Therefore, it is essential to explore strategies that mitigate the effects of this stress. In recent years, seed priming with H_2_S has emerged as an effective chemical strategy to enhance plant tolerance to abiotic stresses, including drought, temperature extremes, UV radiation, and ozone exposure. This protective effect operates through multiple mechanisms: activation of antioxidant defense systems, regulation of stomatal function, optimization of osmotic adjustment, and modulation of stress-responsive gene expression [22,23]. Compared with other priming methods, including hydropriming, osmopriming, and chemical priming with H_2_O_2_, NO, SA, or AsA, H_2_S priming confers deeper biochemical protection through distinct molecular mechanisms. These include protein persulfidation, amplified glutathione–ascorbate redox cycling, and ABA-mediated stomatal regulation, which synergistically minimize ROS accumulation, lipid peroxidation, and cellular damage [23,24,25,26]. Previous research also demonstrated that H_2_S priming improves relative water content, increases antioxidant enzyme activity (SOD, CAT, APX), and leads to osmolytes accumulation such as proline, sugars, glycine betaine [23,27,28,29,30].

Our previous results showed that osmopriming improves *B. napus* (*Brassica napus* L). seed germination and salinity tolerance during post-priming germination and seedling establishment. The improved germination performance was linked with pro-accumulation as a result of H_2_O_2_-induced P5CSA expression and P5CS activity [8]. The initial exposure to osmotic stress created during priming resulted in greater salinity stress tolerance during post-priming germination, a feature likely linked to a “priming memory”. In the study by Lechowska et al. [31] the effect of osmopriming on endogenous polyamine metabolism at the germination and early seedling development of *B. napus* in relation to salinity tolerance were examined. Free, conjugated and bound polyamines were analyzed, and changes in their accumulation were discussed with literature data. The most remarkable differences between the corresponding osmoprimed and unprimed seeds were visible in the free (spermine) and conjugated (putrescine, spermidine) fractions. The arginine decarboxylase pathway seems to be responsible for the accumulation of PAs in primed seeds. The obvious impact of seed priming on tyramine accumulation was also demonstrated. Moreover, salt stress exposure led to a significant increase in ethylene levels in seedlings grown from primed seeds. The authors concluded that the polyamines are involved in creating the beneficial effect of osmopriming on germination and early growth of *B. napus* seedlings under saline conditions through moderate changes in their biosynthesis and accumulation.

However, the role of H_2_S in conferring salinity tolerance in *B. napus* remains insufficiently characterized. Previous research suggests that plants exhibit a dose-dependent response to H_2_S, with micromolar to low-submillimolar concentrations promoting germination and early growth, while higher millimolar concentrations approach toxic thresholds [32].

The aim of this study is to investigate whether seed priming with NaHS, a well-established H_2_S donor, can alleviate salinity-induced oxidative stress and enhance stress tolerance in rapeseed (*Brassica napus* L.). To include both beneficial and potentially inhibitory concentration ranges identified in prior research, two NaHS concentrations were selected: 0.1 mM, representing the upper limit of the growth-promoting range, and 10 mM, situated near the threshold of physiological inhibition. This approach allows comparison of the protective effects of low-dose H_2_S treatment with hydropriming and tests whether *B. napus* exhibits beneficial effects at low H_2_S concentrations but inhibitory effects at high concentrations under saline conditions.

## 2. Results

### 2.1. Impact of Seed Priming on H_2_S Concentration

**Figure 1:**
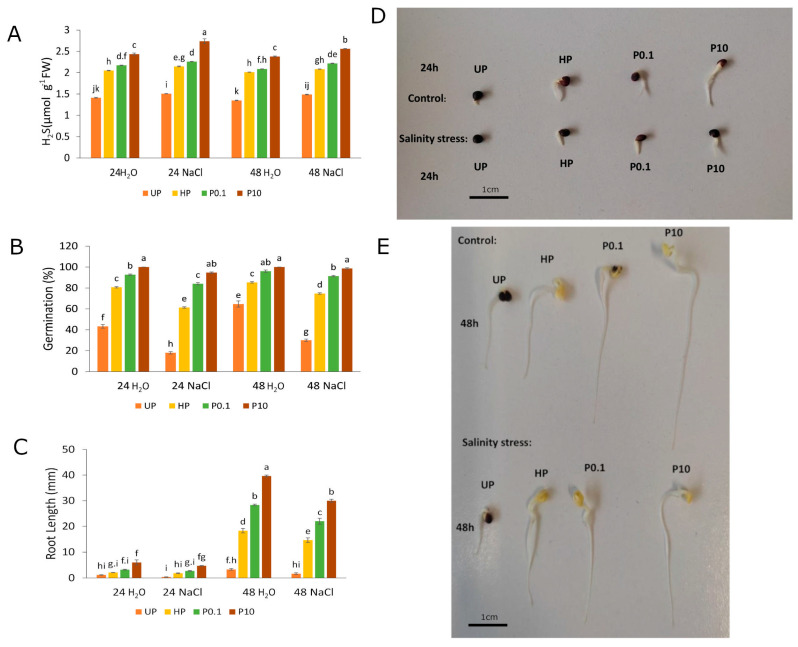
The effect of priming on hydrogen sulfide (H_2_S) level (**A**), germination percentage (**B**) and root length (**C**–**E**) under salinity stress (0.1 M NaCl) after 24 and 48 h of germination as compared to control. Priming treatment: UP (unprimed); HP (hydroprimed); P0.1 (primed with 0.1 mM NaHS (sodium hydrogen sulfide)); P10 (primed with 10 mM NaHS). Means followed by the same letter are not significantly different (*p* < 0.05) by Tukey test. The bars show the standard error of the means (±SE).

During the germination of unprimed seeds (UP) under control conditions, the levels of endogenous H_2_S were similar after 24 h and 48 h, with an average of approximately 1.4 µmol g^−1^ FW (micromoles per gram of fresh weight). Both hydro and NaHS-priming led to an increase in endogenous H_2_S content. In control conditions, the hydropriming of seeds and treatment with lower NaHS concentration led to very similar levels of H_2_S (approximately 2.0 µmol g^−1^ FW); however, application of a higher NaHS concentration (10 mM) resulted in further increase in endogenous H_2_S content (2.4 µmol g^−1^ FW in both 24 h and 48 h time points of germination) (Figure 1A). Germinating seeds exposed to salinity stress were generally characterized by higher H_2_S levels in relation to control conditions. In the germinating UP seeds in both time points (24 h and 48 h), the increase to 1.5 µmol g^−1^ FW in average was observed. In hydroprimed seeds after 24 h of germination, the concentration of endogenous H_2_S was around 2.1 µmol g^−1^ FW on average. The NaHS-priming led to higher endogenous H_2_S levels than hydropriming under salinity stress (Figure 1A). The treatment with 0.1 mM NaHS resulted in average H_2_S concentrations of 2.2 µmol g^−1^ FW after both 24 h and 48 h of germination. The treatment with 10 mM NaHS led to a higher content of endogenous H_2_S during germination in saline conditions. On average, after 24 h of germination, the concentration of 2.7 µmol g^−1^ FW was observed, and after 48 h, a slight but statistically significant decrease occurred (to 2.6 µmol g^−1^ FW on average) (Figure 1A).

### 2.2. Impact of Seed Priming on Germination Percentage

Under non-stress conditions, the germination rate was 43.33% after 24 h and 64.66% after 48 h. After exposure to salinity stress, the germination rate significantly decreased: 18% after 24 h and 30% after 48 h (Figure 1B). Hydropriming moderately improved germination under both conditions, partially mitigating the effects of salinity. After 24 h, hydroprimed seeds showed increased germination rates of 80.66% in water and 61.33% under stress. After 48 h, 85.33% of seeds germinated in water and 74.66% under salinity stress (Figure 1B). Priming with NaHS further enhanced germination. Seeds treated with 0.1 mM and 10 mM NaHS exhibited high germination rates under both control and salinity conditions. After 24 h, treatment with the lower concentration resulted in germination rates of 92.66% in water and 84% under stress (Figure 1B). Germination for 48 h resulted in a further increase in germination rate to 96% in water and 91.33% under salinity conditions. Seeds primed with 10 mM NaHS achieved 100% germination in non-stress conditions at both time points and maintained very high germination rates under salinity stress, with 94.66% under control conditions and 98.66% when exposed to salinity (Figure 1B).

### 2.3. Impact of Seed Priming on Root Length

Root length was also significantly influenced by the priming treatments under both control and salinity stress conditions. UPs exhibited very short root lengths after 24 (Figure 1C,D) and 48 (Figure 1C,E) hours of germination in both salinity and control conditions. However, hydropriming improved root growth under both conditions, particularly after 48 h of germination. Specifically, the root lengths for hydroprimed seeds were 18.33 mm in control conditions and 14.66 mm under salinity stress, compared to 3.33 mm and 1.6 mm for UPs, respectively (Figure 1C,E). Priming with NaHS further enhanced root growth. Germinating seeds pretreated with 0.1 mM NaHS showed root lengths of 28.33 mm after 48 h in control conditions and 22 mm under salinity stress. A similar effect was observed in seeds primed with 10 mM NaHS, achieving during germination root lengths of 39.66 mm in control conditions and 30 mm under salinity treatment. These results demonstrate that NaHS priming significantly promotes root growth and mitigates the inhibitory effects of salinity stress (Figure 1C,E).

### 2.4. Impact of Seed Priming on Oxidative Damage and Total Antioxidant Capacity

**Figure 2:**
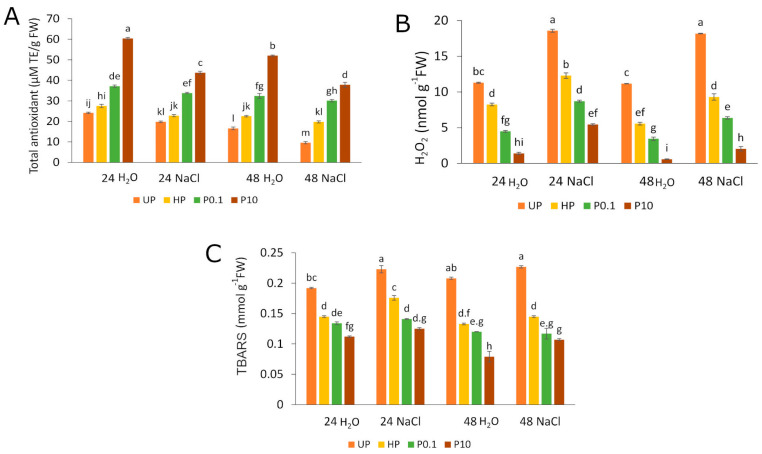
The effect of priming on total antioxidants (**A**), hydrogen peroxide (H_2_O_2_) (**B**), and TBARS (thiobarbituric acid reactive substances) (**C**) under salinity stress (0.1 M NaCl) after 24 and 48 h of germination as compared to control. Priming treatment: UP (unprimed); HP (hydroprimed); P0.1 (primed with 0.1 mM NaHS); P10 (primed with 10 mM NaHS). Means followed by the same letter are not significantly different (*p* < 0.05) by Tukey test. The bars show the standard error of the means (±SE).

In UPs, the highest total antioxidant capacity was observed after 24 h of germination under control conditions (i.e., seedlings grown in water). This value reached 24.12 µM TE/g FW (trolox equivalents/g FW), with the unit representing micromoles of trolox equivalents per gram of fresh weight. After 48 h of germination, this value decreased to 16.57 µM TE/g FW (Figure 2A). Hydropriming induced moderate changes in total antioxidant capacity under control conditions, with values of 27.50 µM TE/g FW after 24 h and 22.52 µM TE/g FW after 48 h (Figure 2A). In contrast, NaHS-priming led to significant increases, particularly at the higher concentration (10 mM), reaching 60.43 µM TE/g FW and 52.03 µM TE/g FW after 24 and 48 h, respectively (Figure 2A). Seedlings germinated from NaHS-primed seeds also maintained significantly higher antioxidant capacity under salinity stress at both time points—43.75 µM TE/g FW after 24 h and 37.83 µM TE/g FW after 48 h. In comparison, UPs exposed to salinity had antioxidant capacities of 19.78 µM TE/g FW after 24 h and 9.62 µM TE/g FW after 48 h of germination. Hydroprimed seeds exposed to salinity showed antioxidant capacities of 22.81 µM TE/g FW and 19.75 µM TE/g FW after 24 and 48 h of germination, respectively (Figure 2A).

The observed changes in antioxidant activity showed a strong inverse correlation with H_2_O_2_ concentrations. Under control conditions, UPs exhibited the highest levels of H_2_O_2_ approximately 11.30 nmol g^−1^ FW after both 24 h and 48 h of germination (Figure 2B), hydropriming resulted in a moderate reduction in H_2_O_2_ content (8.25 nmol g^−1^ FW and 5.56 nmol g^−1^ FW, after 24 h and 48 h, respectively, whereas NaHS-priming led to a significant decrease, particularly at higher concentration of NaHS (1.39 nmol g^−1^ FW after 24 h and 0.585 nmol g^−1^ FW after 48 h). However, even a lower concentration of NaHS (0.1 mM) led to greater decrease in H_2_O_2_ content than hydropriming (4.49 nmol g^−1^ FW and 3.45 nmol g^−1^ FW after 24 h and 48 h, respectively) (Figure 2B). The changes in H_2_O_2_ content during the exposition to salinity stress correlate with changes observed in control conditions. UPs exhibited a concentration of H_2_O_2_ of approximately 18 nmol g^−1^ FW after both 24 h and 48 h of germination. (Figure 2B) Hydropriming led to a decrease in H_2_O_2_ content to 12.29 nmol g^−1^ FW after 24 h and 9.29 nmol g^−1^ FW after 48 h. The treatment with 0.1 mM concentration of NaHS resulted in H_2_O_2_ levels 8.71 nmol g^−1^ FW and 6.73 nmol g^−1^ FW after 24 h and 48 h of germination, respectively, whereas 10 mM concentrations of NaHS resulted in 5.45 nmol g^−1^ FW after 24 h and 2.04 after 48 h (Figure 2B).

Similarly, thiobarbituric acid reactive substances (TBARS) level, which is indicator of lipid peroxidation, was highest in UPs. In the control condition, a concentration of TBARS were approximately 0.2 mmol g^−1^ FW after both 24 h and 48 h of germination (Figure 2C). Hydroprming led to a moderate decrease in TBARS content (to 0.15 mmol g^−1^ FW and 0.12 mmol g^−1^ FW after 24 h and 48 h of germination, respectively). Priming with 0.1 mM of NaHS led to similar TBARS content as hydropriming (with the average 13 mmol g^−1^ FW after 24 h and 0.12 mmol g^−1^ FW after 48 h of germination) while priming with 10 mM NaHS led to lowest TBARS content observed under control condition: 0.112 mmol g^−1^ FW and 0.08 mmol g^−1^ FW after 24 h and 48 h of germination respectively (Figure 2C).

The exposition of UPs to the salinity stress led to increase in TBARS content to 0.22 mmol g^−1^ FW after 24 h of germination and 0.23 mmol g^−1^ FW after 48 h of germination (Figure 2C). In hydroprimed seeds exposed to salinity the TBARS content was lower (0.18 mmol g^−1^ FW after 24 h and 0.16 mmol g^−1^ FW after 24 h of germination) (Figure 2C). Priming with NaHS led to further decrease in lipid peroxidation during exposition to salinity stress. The lower NaHS concentration resulted in an average TBARS content of 0.14 mmol g^−1^ FW after 24 h of stress and 0.12 mmol g^−1^ FW after 48 h. The priming with higher NaHS concentration further decreased the TBARS concentration (to 0.13 mmol g^−1^ FW and 0.11 mmol g^−1^ FW after 24 h and 48 h, respectively (Figure 2C).

### 2.5. Impact of Seed Priming on Cell Membrane Integration

**Figure 3:**
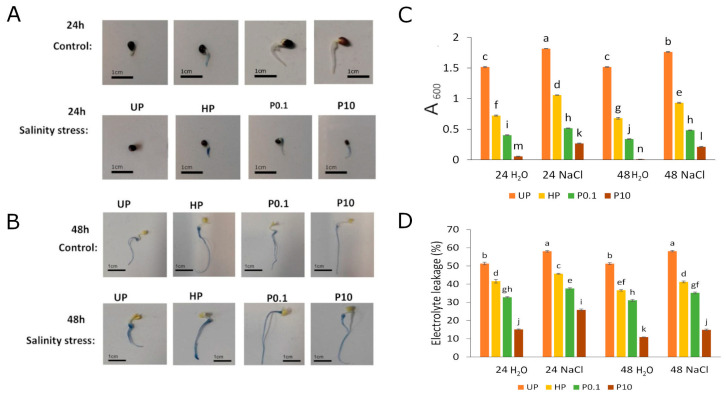
The effect of priming on Evans blue staining (**A**–**C**) and electrolyte leakage (EL) (**D**) under salinity stress (0.1 M NaCl) after 24 and 48 h of germination as compared to control. Priming treatment: UP (unprimed); HP (hydroprimed); P0.1 (primed with 0.1 mM NaHS); P10 (primed with 10 mM NaHS). Means followed by the same letter are not significantly different (*p* < 0.05) by Tukey test. The bars show the standard error of the means (±SE).

In control conditions, the cell membrane integrity, measured with Evans blue staining, significantly increased in all priming conditions (Figure 3A–C). The hydropriming had a strong protective effect on the membrane integrity, however NaHS-priming led to even better results, leading to a further decrease in Evans blue stain uptake proportionally to the applied concentration of NaHS. In both UP and primed seeds, exposure to salinity stress resulted in decreased cell membrane integrity, leading to higher uptake of Evans blue stain. However, the protective effect of priming was maintained (Figure 3A–C). Following 24 h and 48 h of exposure to salinity, hydroprimed seeds were the least protected. In contrast, NaHS-priming resulted in a better protective effect, particularly when a higher concentration of NaHS (10 mM) was applied.

The measurements of electrolite leakage (EL) showed a very good correlation with the results obtained for Evans blue staining. In control conditions, the highest EL was observed in UPs. Hydropriming resulted in a decrease in EL; however, applying both NaHS concentrations further increased cell membrane integrity in a dosage dependent manner (Figure 3C). Exposure to salinity stress led to higher EL compared to control conditions. Similarly, as observed in Evans Blue staining, the protective effect of priming was maintained, with a stronger effect observed for priming with NaHS, especially in 10 mM NaHS (Figure 3C).

## 3. Discussion

Previous studies have reported that salinity stress has a negative effect on germination and growth parameters in plants [4]. Seed germination and seedling growth stages are the most important and most vulnerable stages in the life cycle of plants. Increasing soil salinity reduces the osmotic potential, limiting germination percentage, germination rate and root development Therefore, salinity studies have focused on these two main stages [33]. Recent years have provided an array of studies evidencing the biological effects of H_2_S in plants [14,34,35]. Among them several reports suggested that H_2_S donors stimulated seed germination [36,37]. Nevertheless, these studies essentially pointed the capacity of H_2_S to alleviate the negative effects of stresses on germination, but were poorly informative on the effect of H_2_S on germination *per se*.

The findings of this study strongly support the role of NaHS in seed priming, in enhancing *B. napus* tolerance to salinity stress. In this experimental protocol effects of NaHS priming on seed germination were compared to those caused by hydropriming under control and stress conditions. Hydropriming led to consistent, moderate effects under both control and saline conditions. Specifically, the germination rates of hydroprimed seeds rose markedly compared to those of UP ones, and the root systems were better developed (Figure 1A,B). These results are consistent with previous studies indicating that hydropriming improves germination rate and root growth in *B. napus* [24]. Priming with 0.1 mM NaHS produced even stronger effects, with germination percentages exceeding 90% in water and approximately 84–91% under salinity, along with further improvement in root development as compared to hydropriming (Figure 1A,B). When the NaHS concentration was increased, germination rates climbed to 100% under non-stress conditions and above 94% in salinity. Correspondingly, the main root was the longest observed (Figure 1C). The phenotypic effects correlate well with endogenous H_2_S content, which was lowest in uprimed seeds and the highest in seeds primed with 10 mM NaHS (Figure 1A). Our study showed that seed priming with NaHS significantly alleviated the negative effects of salinity on germination rate and root growth, which is consistent with earlier studies on sunflower [38].

The increased main root length of seedlings grown from NaHS-primed seeds under salinity stress can be attributed to enhanced cytokinesis and cell elongation in roots, both of which are promoted by H_2_S. Studies have shown that H_2_S stimulates mitotic activity in root meristems by upregulating key cell cycle regulators such as cyclins and CDKs, as demonstrated in rice [39] and maize [40]. Additionally, H_2_S enhances cell elongation by modulating auxin signaling and promoting the expression of cell wall-loosening enzymes, as observed in *Arabidopsis* and tomato [41,42]. These coordinated effects on cell division and expansion contribute to improved root architecture during germination, especially under stress conditions.

One crucial mechanism evolved by plants to adapt to salinity stress includes the induction of enzymatic and non-enzymatic antioxidants, which are important for eliminating ROS and maintaining cellular redox potential [14,34,35]. The increased germination rate and root length of rapeseed (*Brassica napus* L.) may be explained, at least in part, by changes in total antioxidant capacity. This capacity was slightly increased in hydroprimed seeds, increased further in seeds primed with 0.1 mM NaHS, and was highest in seedlings from the 10 mM NaHS treatment (Figure 2A). An increase in total antioxidant capacity was associated with a lower H_2_O_2_ concentration (Figure 2B) and, consequently, reduced TBARS content (Figure 2C), suggesting a protective effect against ROS generation and lipid oxidative damage. This protective effect was observed with hydropriming and was further enhanced by NaHS-priming, with the lowest H_2_O_2_ and TBARS levels observed after treatment with 10 mM NaHS. The observed increase in total antioxidant capacity led to a lower H_2_O_2_ concentration and, consequently, to reduced TBARS content, indicating a protective role of priming against reactive oxygen species (ROS) generation and lipid oxidative damage. Furthermore, H_2_S has been shown to efficiently improve seed germination under salinity stress by modulating the antioxidant system and cellular redox balance [28]. The protective effect against oxidative damage is further supported by changes in Evans Blue staining (Figure 3A–C) and EL measurements (Figure 3D), in which hydropriming produced a moderate decrease in both parameters. In contrast, the strongest effect of membrane stabilization was observed after priming with 10 mM NaHS. These changes can be interpreted as potential causes for the observed improvement in the germination of the primed seeds, allowing for the reduction in early imbibitional damage and improved reorganization of the membranes. The protective effect of H_2_S against oxidative damage under saline conditions, including protection of cellular structures and enhancement of seed viability, was also observed in *Medicago sativa* [43]. Research on *Hordeum vulgare* [44] and *Triticum aestivum* [45] also indicates that H_2_S alleviates the effects of salinity by maintaining lower intracellular sodium concentrations. Additionally, NaHS pretreatment mitigates salinity stress by increasing the activity of hydrolytic enzymes such as amylase [46], which is essential for mobilizing stored nutrients during germination. Amylase breaks starch into soluble sugars, supplying energy for embryo development, while protease degrades storage proteins into amino acids, supporting cellular synthesis and growth. NaHS is a widely used H_2_S donor in seed priming due to its affordability, water solubility, and rapid release of bioactive H_2_S upon dissolution. These characteristics make it particularly effective for short-term priming to promote seed germination and early seedling vigor under abiotic stress. Compared to other H_2_S donors, NaHS offers a practical balance of efficiency and accessibility. Notably, NaHS has been shown to induce faster antioxidant responses in *Arabidopsis* under cadmium stress than slow-releasing donors such as GYY4137 [47]. GYY4137, on the other hand, offers sustained H_2_S release, beneficial for long-term stress studies, but comes at a higher cost and is less accessible. Another popular H_2_S donor, Na_2_S, may cause pH shifts [48,49]. In turn, Lawesson’s reagent and DADS (diallyl disulfide) issues with solubility, specificity, or toxicity, limiting their practicality [22].

Surprisingly, no toxicity effect was observed at 10 mM NaHS, despite this concentration being near the threshold of physiological inhibition. This result indicates that *B. napus* seeds may possess mechanisms that buffer or restrict the effective uptake of H_2_S, thereby preventing internal concentrations from reaching damaging levels even at high external doses. Factors such as limited seed coat permeability or metabolic conversion of H_2_S, and efficient detoxification pathways may help maintain intracellular H_2_S within a tolerable range. Additionally, germinating seeds may temporarily increase their capacity to utilize H_2_S as a signaling molecule rather than a stressor, potentially shifting the toxicity threshold upward under these conditions. Collectively, these mechanisms may account for the absence of inhibitory effects at 10 mM NaHS.

Beyond H_2_S donors, NaHS-priming has been compared to other priming techniques. Osmopriming with PEG lacks the signaling specificity of H_2_S [50]. Hormonal priming activates targeted pathways but is often species-dependent and cost-intensive [51]. Nano-priming, using nanoparticles like ZnO or SiO_2_, enhances stress tolerance and nutrient uptake but raises concerns about environmental safety and scalability [52]. In contrast, NaHS-priming is simple, scalable, and mechanistically robust. Importantly, other investigations involving hydropriming have also demonstrated improvements in germination and seedling vigor under moderate stress conditions [53]. These findings align with our results. NaHS stands out by its ability to activate multiple stress-responsive signaling pathways, particularly those involved in antioxidant defense, osmotic regulation, and root system development. Exogenous H_2_S released from NaHS enhances the activity of key antioxidant enzymes such as SOD, CAT, and APX, and modulates redox-related transcriptional networks, consequently strengthening cellular protection against salinity-induced oxidative stress [37,42]. Concurrently, H_2_S signaling facilitates root growth and architectural remodeling through interactions with auxin, nitric oxide, and calcium signaling pathways, which enables seedlings to more efficiently explore the soil and maintain ion homeostasis under saline conditions [35,54]. These integrated physiological and molecular responses make NaHS-priming a more dynamic and efficient strategy when compared to conventional osmotic or nutrient-based priming methods, supporting its application in both research and agronomic practice. In addition to its biological efficiency, NaHS offers several practical benefits: it is cost-effective, easy to prepare, and compatible with standard seed treatment equipment, enabling straightforward incorporation into existing priming protocols without significant procedural modifications. Collectively, these characteristics place NaHS priming as a viable and scalable approach for improving seedling growth and stabilizing crop yields in saline agricultural conditions.

## 4. Materials and Methods

### 4.1. Seed Sterilization and Priming Procedure

Seeds of *Brassica napus* L. cv Libomir were surface-sterilized by immersion in a 15% sodium hypochlorite (NaOCl) solution for 5 min, followed by five washings with distilled water.

For the seed priming treatments, 2 g of seeds were soaked in 1 mL of solution on Petri dishes under two conditions:

- Hydropriming using distilled water (H_2_O)
- Priming using NaHS at two concentrations—0.1 mM (P0.1) and 10 mM (P10)

The priming was conducted in the dark at 25 °C for 48 h. After priming, the seeds were removed from the solutions, thoroughly rinsed with distilled water, and dried at 25 °C for 48 h to restore their original moisture content and match the weight of UPs. The experiment was conducted using a completely randomized design with three replications per treatment. Each replication consisted of 50 seeds placed in a Petri dish. Both UP and primed seeds were germinated on two layers of sterile, moist filter paper within Petri dishes, which were maintained in a dark room at 25 °C. To simulate control and salinity stress conditions, seeds were germinated either on distilled water or on 0.1 M NaCl solution. To assess germination performance and early biochemical changes, measurements were taken at two-time intervals: 24 h and 48 h after starting the experiment.

### 4.2. Germination Percentage and Root Length

To evaluate the germination dynamics of primed and UPs under control and saline conditions after 24 and 48 h from the start of the experiment, the final germination percentage (FGP) was calculated, with germination defined as visible radicle emergence, according to the equation:

$$FGP=\frac{\mathrm{the}\;\mathrm{final}\;\mathrm{count}\;\mathrm{of}\;\mathrm{germinated}\;\mathrm{seeds}}{\mathrm{the}\;\mathrm{total}\;\mathrm{count}\;\mathrm{of}\;\mathrm{seeds}\;\mathrm{at}\;\mathrm{the}\;\mathrm{beginning}}\;\begin{array}{cc} \times & 100 \end{array}$$

A caliper with an accuracy of one-tenth of a millimeter was used to measure the root length.

### 4.3. H_2_O_2_ Concentration

To quantify H_2_O_2_ levels, 0.5 g of the whole *B. napus* early seedlings were homogenized in 6 mL of 0.1 M phosphate buffer (pH 7.8) containing 0.05 g of activated charcoal [55]. Activated charcoal was included to remove interfering phenolic compounds that could affect the accuracy of the colorimetric assay, although its use may also lead to partial degradation of H_2_O_2_ and thus lower measured values [56].

The homogenate was centrifuged at 15,000× *g* for 30 min at 4 °C. For the assay, 1000 μL of phosphate buffer, 667 μL of the supernatant (tissue extract), and 833 μL of a pre-prepared titanium reagent were mixed in a spectrophotometric cuvette. The mixture was incubated at room temperature for 10 min. Absorbance was then measured at 508 nm using a spectrophotometer (Ultrospec 4000 UV/Vis Spectrophotometer, Pharmacia Biotech, Uppsala, Sweden). The concentration of H_2_O_2_ was calculated based on a standard calibration curve prepared using known concentrations of H_2_O_2_ [55].

### 4.4. Lipid Peroxidation

To assess lipid peroxidation, 0.25 g of whole *B. napus* early seedlings was homogenized in 5 mL of distilled water. A solution containing 0.5% thiobarbituric acid (TBA) in 20% trichloroacetic acid (TCA) was prepared. Then, 5 mL of the TBA-TCA solution was added to the homogenate, thoroughly mixed, and transferred into 15 mL Falcon tubes.

The samples were incubated in a water bath at 95 °C for 30 min to allow the formation of the malondialdehyde -TBA and other aldehyde-TBA complexes. After incubation, the tubes were cooled to room temperature and then centrifuged at 12,000 rpm for 15 min at 20 °C.

The absorbance of the supernatant was measured at 532 nm and 600 nm using a spectrophotometer (Ultrospec 4000 UV/Vis Spectrophotometer, Pharmacia Biotech, Uppsala, Sweden). The absorbance at 600 nm was subtracted from that at 532 nm to correct for non-specific absorbance. The TBARS content was calculated using the extinction coefficient [57].

### 4.5. Electrolyte Leakage (EL)

The *B. napus* early seedlings were collected from each experimental treatment, and its FW was recorded. The samples were gently washed with distilled water to remove surface contaminants. Each sample was then placed into a 50 mL Falcon tube containing 40 mL of distilled water and incubated at room temperature for 2.5 h. After incubation, the initial electrical conductivity (EC_1_) of the solution was measured using a conductivity meter (CC-411, Elmetron, Zabrze, Poland). The tubes were then placed in a boiling water bath for 30 min to release all electrolytes. After boiling, the samples were cooled to room temperature on ice, and the final electrical conductivity (EC_2_) was measured.

EL was calculated as the ratio of EC_1_ to EC_2_ and expressed per gram of FW using the following formula [58]:

$$EL\;(\%)=\frac{{EC}_{1}}{{EC}_{2}}\times 100$$

### 4.6. Evans Blue Staining

Evans blue staining was performed using early *B. napus* seedlings (one seedling per sample). After gentle washing, each seedling was dried, weighed, and its FW was recorded. Seedlings (typically 20–40 mg FW each) were placed individually into 15 mL Falcon tubes containing 10 mL of Evans blue solution (0.25 g Evans blue in 250 mL distilled water) and incubated for 10 min. The Evans blue solution was poured into a beaker. The plants were washed with water 3 times. An observation was made, and a photo was taken. 50% methanol was prepared. 1 g of sodium dodecyl sulfate (SDS) was dissolved in 100 mL of 50% methanol. Plant tissue was homogenized in SDS in methanol using the proportion of 1 g of plant tissue to 20 mL of extracting solution. The same amount of plant materials was taken. The homogenate was incubated for 30 min at 50 °C. The plant extract was centrifuged at 7000× *g* for 5 min. The absorbance value of the supernatant was measured at 600 nm (Ultrospec 4000 UV/Vis Spectrophotometer, Pharmacia Biotech, Uppsala, Sweden). The results were expressed as an absorbance value [59].

### 4.7. Determination of Total Antioxidant Activity (DPPH Assay)

To assess total antioxidant activity, 24 mg of 2,2-diphenyl-1-picrylhydrazyl (DPPH) was dissolved in 100 mL of methanol to prepare the stock solution. A working solution was then prepared by diluting 10 mL of the stock with 45 mL of methanol, and the absorbance was adjusted to 1.10 ± 0.01 at 517 nm.

For sample preparation, 0.25 g of fresh plant tissue was homogenized in 5 mL of methanol. The homogenate was centrifuged at 10,000 rpm for 20 min at 4 °C. Then, 150 µL of the resulting supernatant was mixed with 2850 µL of the DPPH working solution. The mixture was incubated in the dark at room temperature for 30 min.

After incubation, the absorbance was measured at 517 nm using a spectrophotometer (Ultrospec 4000 UV/Vis Spectrophotometer, Pharmacia Biotech, Uppsala, Sweden). Total antioxidant activity was calculated based on a standard calibration curve [60].

### 4.8. Determination of H_2_S Content

To quantify endogenous H_2_S levels, 0.25 g of whole *B. napus* early seedlings was ground in liquid nitrogen. One milliliter of extraction buffer (20 mM Tris-HCl buffer (pH 8.0) containing 10 mM EDTA and 20 mM Zn (OAc)_2_) was added to the powdered tissue, and the mixture was homogenized thoroughly. The homogenate was then centrifuged at 15,000× *g* for 15 min at 4 °C.

For the assay, 200 μL of the supernatant was mixed with 3760 μL of extraction buffer and 40 μL of 5,5-dithiobis (2-nitrobenzoic acid) (DTNB) reagent. The reaction mixture was incubated in the dark at room temperature for 2 min. Absorbance was measured at 412 nm using a spectrophotometer (Ultrospec 4000 UV/Vis Spectrophotometer, Pharmacia Biotech, Uppsala, Sweden).

The concentration of H_2_S was determined by comparing the absorbance (A_412_) to a standard calibration curve prepared using known concentrations of NaHS [61].

### 4.9. Statistical Analysis

All statistical analyses were performed using data from at least three independent experiments. Differences among priming treatments (unprimed, hydroprimed, NaHS 0.1 mM, and NaHS 10 mM) within each salinity condition and time point were evaluated using one-way analysis of variance (ANOVA). When ANOVA indicated significant effects, mean separation was conducted using the Tukey–Kramer post hoc test. All analyses and graph preparation were carried out in OriginLab (OriginPro, OriginLab Corporation, Northampton, MA, USA). Statistical significance was accepted at *p* < 0.05.

## 5. Conclusions

Although hydropriming provides moderate protection against salinity in *B. napus*, the NaHS treatment, particularly at higher concentrations, is more effective in enhancing its resilience to soil salinization. As treatments with H_2_S donors efficiently alleviate inhibition of germination by salinity stress, future works should be focused on deciphering whether endogenously evoked H_2_S participates in salinity stress tolerance during seed germination and could therefore constitute a trait for variety selection and improvement. Altogether, the exact mechanisms of H_2_S induced salinity tolerance remain to be elucidated using molecular, physiological, biochemical, omics, and multiomics approaches. Moreover, the interaction between H_2_S and other signaling molecules should stand for future in-depth research on the mechanism of action of H_2_S.

## Data Availability

The original contributions presented in this study are included in the article. Further inquiries can be directed at the corresponding author.

## Abbreviations

The following abbreviations are used in this manuscript:

APX	ascorbate peroxidase
CAT	catalase
CO	carbon monoxide
DPPH	2,2-diphenyl-1-picrylhydrazyl
DTNB	5,5-dithiobis (2-nitrobenzoic acid)
EC	electrical conductivity
EL	electrolite leakage
FGP	final germination percentage
FW	fresh weight
H_2_O_2_	hydrogen peroxide
H_2_S	hydrogen sulfide
HP	hydropriming
NaHS	sodium hydrogen sulfide
NaOCl	sodium hypochlorite
NO	nitric oxide
SDS	sodium dodecyl sulfate
SOD	superoxide dismutase
TBA	thiobarbituric acid
TBARS	thiobarbituric acid reactive substances
TCA	trichloroacetic acid
TE	trolox equivalents
UP	unprimed
Zn (OAc)_2_	zinc acetate
